# *Meloidogyne enterolobii* egg extraction in NaOCl versus infectivity of inoculum on cucumber

**DOI:** 10.21307/jofnem-2021-057

**Published:** 2021-06-22

**Authors:** Guillermo Gómez-González, Isabel Cruz-Lachica, Isidro Márquez-Zequera, José Benigno Valdez-Torres, Juan Manuel Tovar-Pedraza, Luis Alfredo Osuna-García, Raymundo Saúl García-Estrada

**Affiliations:** 1 Centro de Investigación en Alimentación y Desarrollo, A. C. Carretera Culiacán-Eldorado, km 5.5. Culiacán, Sinaloa 80110, México

**Keywords:** Cucumber, Egg extraction, Hatching, Infectivity, Interaction, *Meloidogyne enterolobii*, NaOCl, Pathogenicity, RKN, Stirring

## Abstract

Extraction of eggs of *Meloidogyne* spp. in sodium hypochlorite (NaOCl) is a helpful procedure to assess the population levels and to obtain inoculum. In this sense, laboratory and greenhouse experiments were done to evaluate the effect of the NaOCl concentration on the viability of *M. enterolobii* eggs. Additionally, the objective of this investigation was to corroborate the preferable treatments to count populations in cucumber galled roots or to obtain inoculum of *M. enterolobii.* It was shown that the effect of the NaOCl concentration on the viability of *M. enterolobii* eggs is potentially detrimental. The NaOCl concentration caused a higher hatching, which in turn, resulted in non-infective larvae. Therefore, the best treatments to obtain inoculum of eggs of *M. enterolobii* included the 0.75% NaOCl (with 8-min stirring), 0.5% NaOCl (with stirring for 8, 12, and 16 min), and 0.3% NaOCl concentration (with stirring for 8, 12, 16, and 20 min). For a correct estimate of the egg population in roots, we show by several treatments that a concentration of 0.5% NaOCl (with stirring for 8, 12, and 16 min) and 0.75% NaOCl (with 8-min stirring) give the highest results.

The root-knot nematode (RKN) *Meloidogyne enterolobii* Uang and Eisenback is a widely dispersed and highly pathogenic plant parasite. This RKN is capable for reproduction on most of vegetables, including cultivars with genetic resistance to other important RKN species such as *M. incognita* and *M. javanica* (Brito et al., 2004). In Mexico, *M. enterolobii* was recently diagnosed by molecular identification in the vegetable-producing region of Sinaloa, parasitizing several crops including tomato, pepper, and cucumber (Gómez-González et al., 2020). The yield loss caused by RKN depends on population levels in soil including the eggs on residual root systems from previous crops. Therefore, the extraction and quantification of the actual inoculum density constitutes part of the management of RKN (Greco and Di Vito, 2009). Currently, control strategies such as plant resistance or biological control are being actively investigated for *M. enterolobii* (Castagnone-Sereno, 2012). These modern experimental studies involve classical nematological techniques such as the extraction of eggs via NaOCl method (Hussey and Barker, 1973). This technique helps to assess the RKN populations and to obtain specimens and large amounts of inoculum.

Sodium hypochlorite in water reacts to form the hypochlorous acid (HClO) that causes oxidative degradation of microbial cell structures upon contact, also, NaOCl damages vegetable tissues at concentrations above 200 ppm (Mishra et al., 2018). In nematology, NaOCl has been employed as a standard technique for RKN egg extraction. Initially, NaOCl was employed to dissolve egg masses of *M. incognita* from cucumber galled roots by grinding for 40 sec in 20% NaOCl solution (McClure et al., 1973). Simultaneously, treatments involving different concentrations (2.62, 1.05, and 0.53%) of NaOCl and vigorous shaking of tomato roots for 4 min were studied. It was determined that concentrations of 1.05 and 0.53% resulted in inoculum capable to infect roots (Hussey and Barker, 1973). However, it was also suggested that 0.53% NaOCl caused a hatching delay and therefore, a lower infectivity (19.8%) when compared with intact egg masses (51.4%) (Vrain, 1977). Similarly, egg viability from dissolved egg masses in 1.0% NaOCl has been described as significantly lower (12%) when compared with viability from egg masses in water (58%) (Di Vito et al., 1986). Afterward, a comparison between stirring and grinding of 1.5 g of tomato and soybean roots (2.5-cm pieces) in NaOCl solution of 0.5%, showed that the stirring treatment for 10 min provides a most efficient extraction of *M. incognita* eggs, compared with grinding (Stetina et al., 1997).

In recent years, different researchers have used the NaOCl method for different purposes, one example is to extract inoculum directly (Dong et al., 2007; Maleita et al., 2012; Thomas et al., 1997; Vovlas et al., 2005a, b). Another implementation of the NaOCl method has been to obtain J2 from extracted eggs (Faske and Hurd, 2015; Giné et al., 2014; Kayani et al., 2016). Also, the NaOCl method has been employed to quantify the reproduction from an inoculated population (Djian-Caporalino et al., 2011; Giné et al., 2017; Kayani et al., 2018; Vovlas et al., 2005b). Unfortunately, the impact of this protocol on the viability of the unhatched juvenile is usually omitted. On the other hand, the procedures used for egg extraction and RKN species vary among studies. Therefore, data on the effects of the NaOCl method on extraction and infectivity of *M. enterolobii* will contribute to reduce variability in evaluations employing this technique. The objective of this investigation was to describe the effect of the NaOCl concentration on the viability of *M. enterolobii* eggs. Additionally, the objective of this investigation was to corroborate the preferable NaOCl-stirring treatments to inoculum of *M. enterolobii.*

## Materials and methods

### Extraction tests

Cucumber (*Cucumis sativus* L. ‘Espada’) seeds were established in infested soil with a previously isolated local strain of *M. enterolobii* (Gómez-González et al., 2020). The plants were allowed to grow for two months in a greenhouse. Then, the galled roots were washed, extracted, cut (1.5-cm segments), and mixed in clean tap water for tissue homogenization. After a short drain, root samples of 20 g were handpicked for each extraction. The treatments were established by factorial arrangement with four NaOCl concentrations and four stirring periods. For this purpose, a solution of sodium hypochlorite 6% a.i. (ICR, Mexico) was diluted adding distilled water (pH 7.0) to obtain the final NaOCl concentrations of: 0.3, 0.5, 0.75, and 1.0%. The stirring was performed with a Hanna HI322 (Smithfield, RI) magnetic stirrer (400 rpm) in 500-ml flasks. The stirring periods were: 8, 12, 16, and 20 min. After stirring, dilutions were poured onto nested sieves of 75, 45, and 25-µm-pore mesh and washed several times with distilled water to remove residues of NaOCl. Eggs were collected in sterilized tap water and counted to evaluate the extraction level. Immediately after counting, eggs from each replication were used as inoculum in the further tests. The experimental design for extraction tests was completely randomized with six replications.

### Hatching tests

A layer of Kimwipe tissue was set on nylon sieves in trays filled with sterilized tap water (Modified Baermann’s Technique). Aliquots from each replication of extraction treatments containing 1,000 ± 10 eggs were spread on the Kimwipe tissue to allow the hatching and migration of J2. Nylon sieves with one-gram samples of galled tissue were used as a control. Trays were placed in a complete randomized design with six replications in the laboratory at 26°C for five days (Hooper et al., 2005). Larvae were collected daily and counted.

### Reproduction tests

Cucumber seedlings cv. Espada of two weeks after planting (WAP) were individually transplanted in 1.6-liter pots filled with steam-pasteurized (100°C × 45 min) silty clay loam soil (7% sand, 60% silt, 33% clay; pH 6.8). Two holes of 4-cm deep and 1-cm wide were made in the soil surrounding each transplanted seedling with the use of a pipette. Then, each pot was inoculated with a 10-ml aliquot containing 1,500 ± 15 eggs from each replication of extraction treatments. Inoculation with 300 ± 6 J2 was used as control (Dong et al., 2007). Pots were set in a randomized complete block design with six replications. Seedlings were allowed to develop for five weeks after inoculation (WAI) in a greenhouse at 21 to 33°C. Pots were watered daily. Finally, roots were washed in tap water, extracted, drained briefly, and then each complete root system was stirred for 10 min with 0.5% NaOCl to determine the production of eggs (Stetina et al., 1997).

### Infectivity tests

Two cucumber seedlings of three WAP were transplanted in 15-liter pots filled with soil as described above. Then, each pot was inoculated with a 20-ml aliquot containing 6,000 ± 30 eggs from each replication of extraction treatments. Inoculation with water or 1,200 ± 12 J2 were used as negative and positive controls (Dong et al., 2007). A randomized complete block design with four replications was used. Seedlings were allowed to develop for 17 WAI in a greenhouse at 14 to 29°C and watered daily. Later, the cucumber roots were extracted and washed. Galling percentages were visually considered comparing the root systems from egg treatments against healthy root systems from control treatment. Finally, roots were cut and samples were picked to determine the quantities of eggs per gram of root, as described above. All experiments were repeated twice under similar conditions.

### Statistical analysis

Prior to statistical analysis, data from both trials of each experiment were combined. Then, data from egg counts were ln transformed, and data from hatched J2 were first adjusted by subtracting the average amount of J2 obtained in the control and then √ transformed. Data were analyzed using the Proc GLM for a two-way ANOVA to determine the significance of both factors. A Fisher’s least significant difference (LSD) test (*α* = 0.05) was used to determine main-effect means (SAS v.9.1) (SAS Institute, Cary, NC). In the same way, a Pearson’s correlation coefficient test was used to establish relationships between the number of produced eggs and hatched J2. Additionally, these data were also analyzed by regression with numbers of hatched J2 as the independent variable (SigmaPlot v.14) (Systat Software, San Jose, CA).

## Results

### Extractions tests

**Figure 1:**
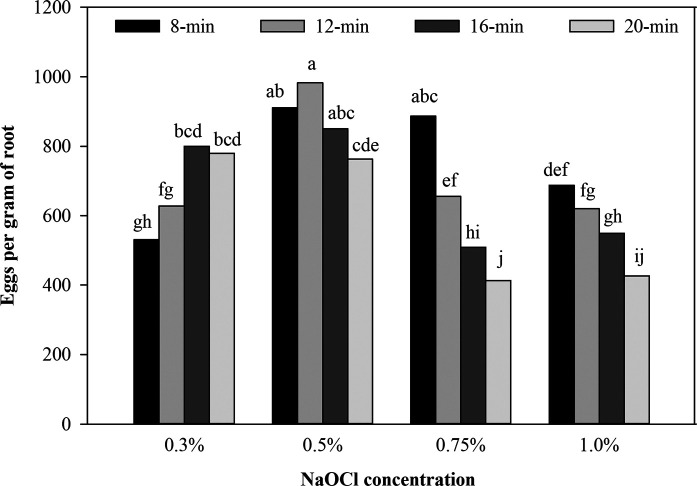
Effect of combinations of NaOCl concentrations and stirring periods on *M. enterolobii* egg extraction from cucumber roots. Data are means of 12 replications (six replications/trial). Bars with the same letter are not significantly different (*p >* 0.05) by Fisher’s least significant difference (LSD) test.

At low NaOCl concentration (0.3%) with stirring for 16 to 20 min the extraction level was higher (*p ≤ *0.05) when compared with stirring for 8 to 12 min. At NaOCl concentration of 0.5% the level of extracted eggs was similar (*p >* 0.05) among stirring periods for 8 to 16 min. However, at 0.75% NaOCl only the stirring period of 8 min observed a similar (*p > *0.05) extraction level when compared with the 0.5% NaOCl treatments. The lower (*p ≤* 0.05) extraction level was observed with treatments of 0.3% NaOCl with stirring for 8 to 12 min and 0.75 to 1.0% NaOCl with stirring for 12 to 20 min (Fig. 1).

### Hatching tests

**Figure 2:**
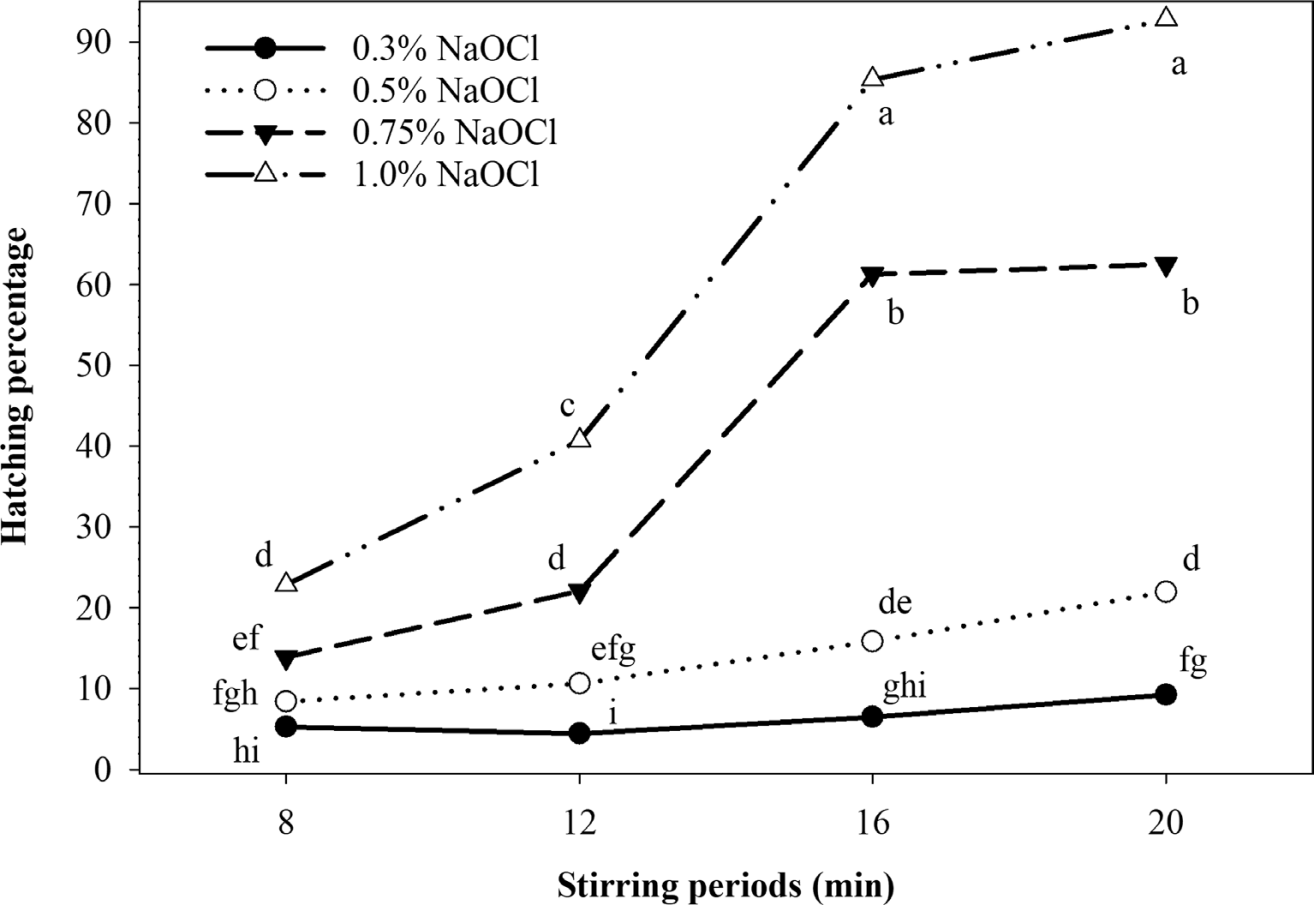
Effect of NaOCl concentrations and stirring periods on *M. enterolobii* hatching. Data are means of 12 replications (six replications/trial). Symbols with the same letter are not significantly different (*p > *0.05) by Fisher’s least significant difference (LSD) test.

The combinations of the NaOCl concentrations 0.75 and 1.0% with stirring periods for 16 to 20 min resulted in a higher (*p ≤* 0.05) induction of hatching when compared with the remaining treatments (Fig. 2). This effect varied from 61 to 93% of hatched larvae. The 0.5 and 0.3% NaOCl treatments showed a shorter range of hatching that varied from 5 to 22%. It is worth noting that the induced hatching level from all NaOCl treatments combined with the stirring period of 8 min also varied in a range from 5 to 23%. The 0.3% NaOCl treatments induced a lower (*p ≤* 0.05) hatching level when compared with the rest of NaOCl treatments within each stirring period for 12 to 20 min (Fig. 2).

### Reproduction tests

**Figure 3:**
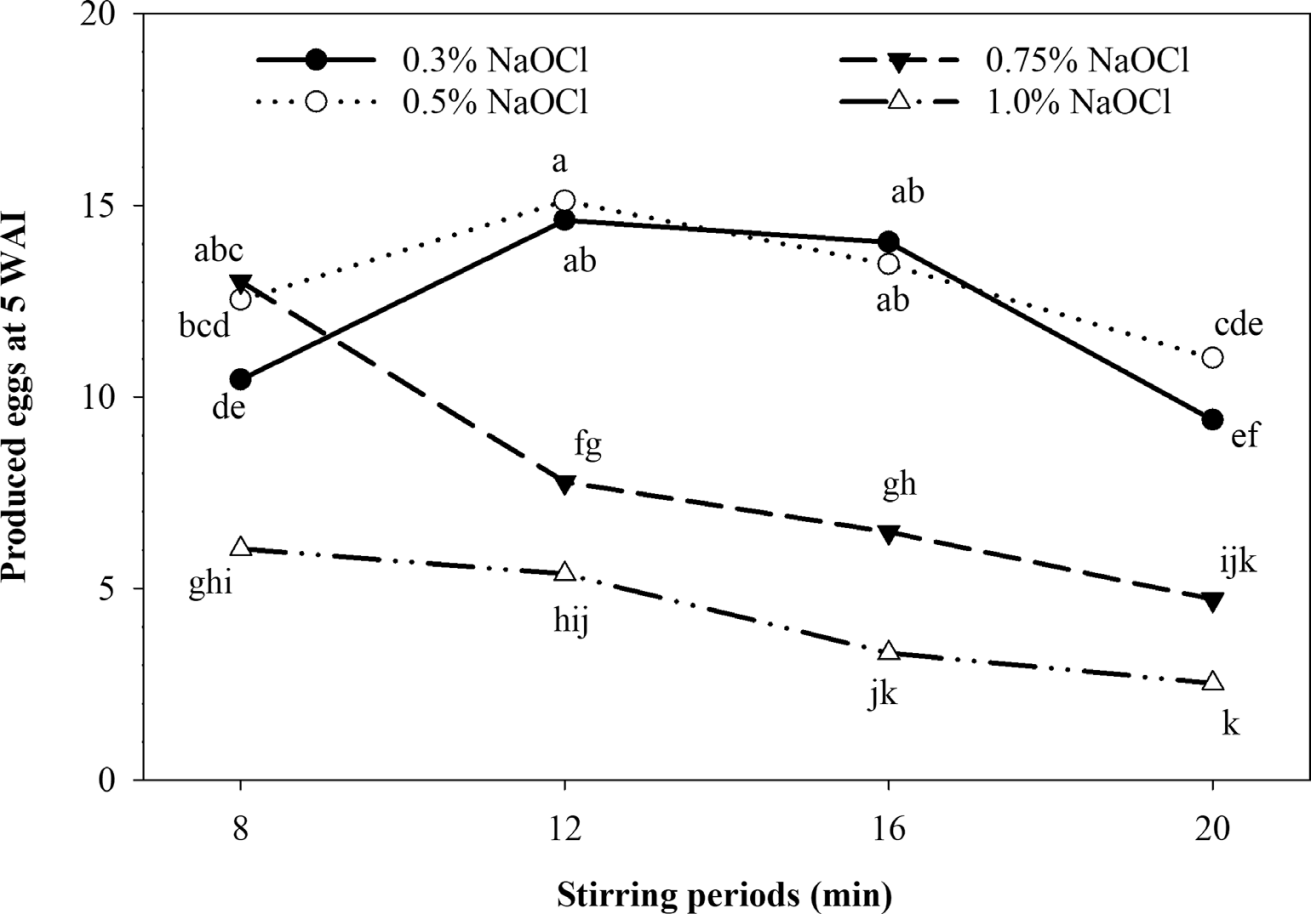
Effect of NaOCl concentrations and stirring periods on *M. enterolobii* reproduction. Data are means of 12 replications (six replications/trial). Symbols with the same letter are not significantly different (*p >* 0.05) by Fisher’s least significant difference (LSD) test. Numbers of produced eggs are presented in thousands.

**Figure 4:**
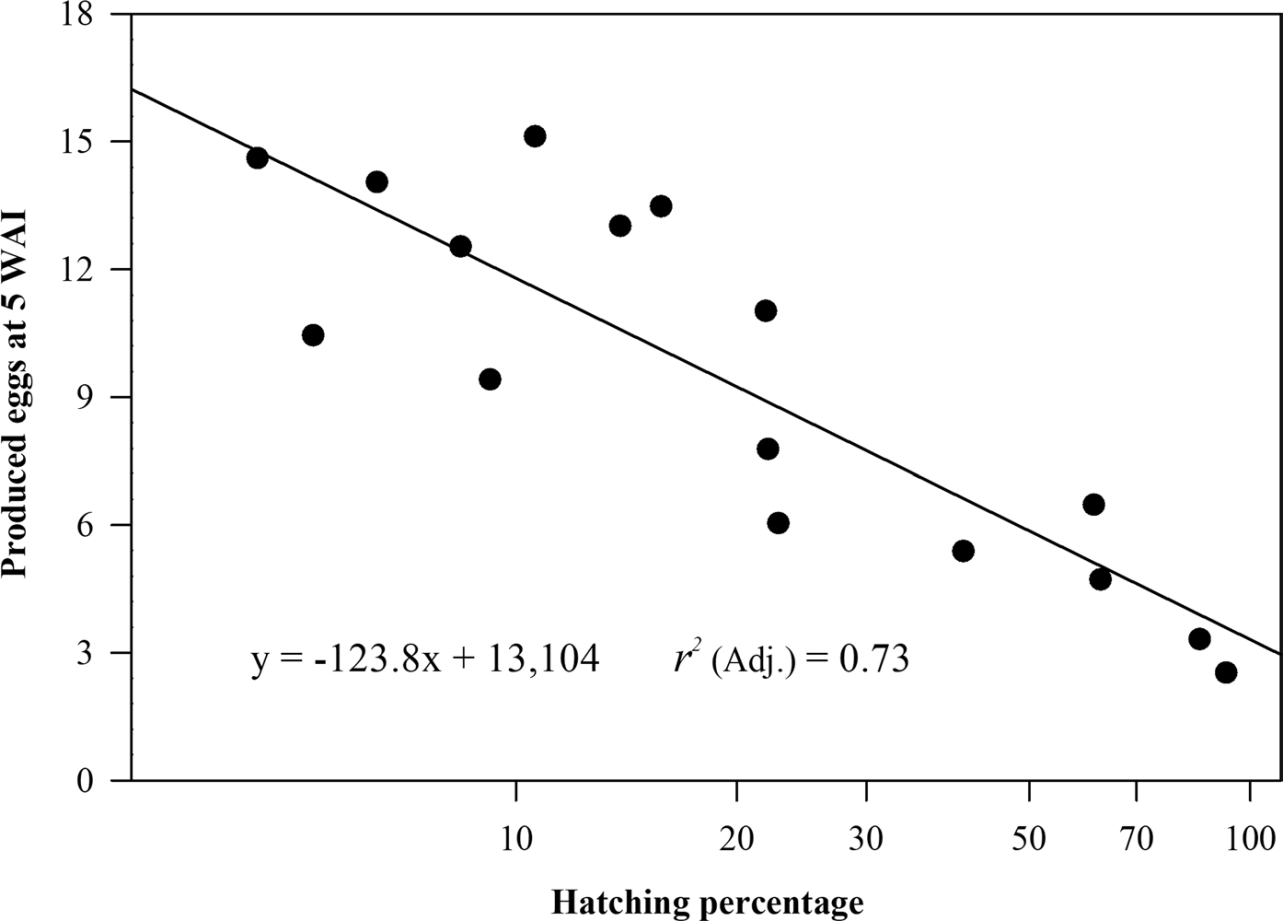
Effect of *M. enterolobii* hatching on inoculum reproduction. Equation derived from linear regression (*p ≤* 0.05). Inoculum reproduction is presented in thousands of eggs.

In the reproduction tests at 5 WAI, larvae treatment (control) resulted in a higher count of 16,836 eggs per root system, when compared with eggs as inoculum (*p ≤* 0.05) (data not shown). Importantly, although the weight of the cucumber root systems (concomitant variable) at 5 WAI varied from 10 to 22 g among treatments (data not shown), the extraction level of *M. enterolobii* eggs was unaffected (*p >* 0.05). On the other hand, at 17 WAI the extraction of eggs from samples was useless to estimate the population levels from whole root systems. However, in the reproduction tests at 5 WAI, the treatment of 0.75% NaOCl with stirring for 8 min observed a higher (*p ≤* 0.05) reproduction level when compared with the treatments with 0.3% NaOCl with stirring for 8 and 20 min (Fig. 3). Also, the 0.75% NaOCl with stirring for 8 min observed a higher (*p ≤* 0.05) reproduction level when compared with all 1.0% NaOCl treatments and 0.75% NaOCl (12 to 20-min stirring). The 0.75% NaOCl with stirring for 8 min resulted in a similar (*p >* 0.05) reproduction level compared to all 0.5% NaOCl treatments. On the other hand, the reproduction level with the 0.3 and 0.5% NaOCl treatments was similar (*p >* 0.05) within each stirring period. The 1.0% treatments showed the lower (*p ≤* 0.05) reproduction levels within stirring periods for 8 to 16 min. With the stirring period of 20 min, the reproduction level observed was similar (*p >* 0.05) between treatments of 0.75 and 1.0% NaOCl (Fig. 3). Finally, the regression analysis showed a negative correlation coefficient (*R* = ‒0.86); i.e., the production of eggs decreased as the hatching increased (Fig. 4).

### Infectivity tests

**Figure 5:**
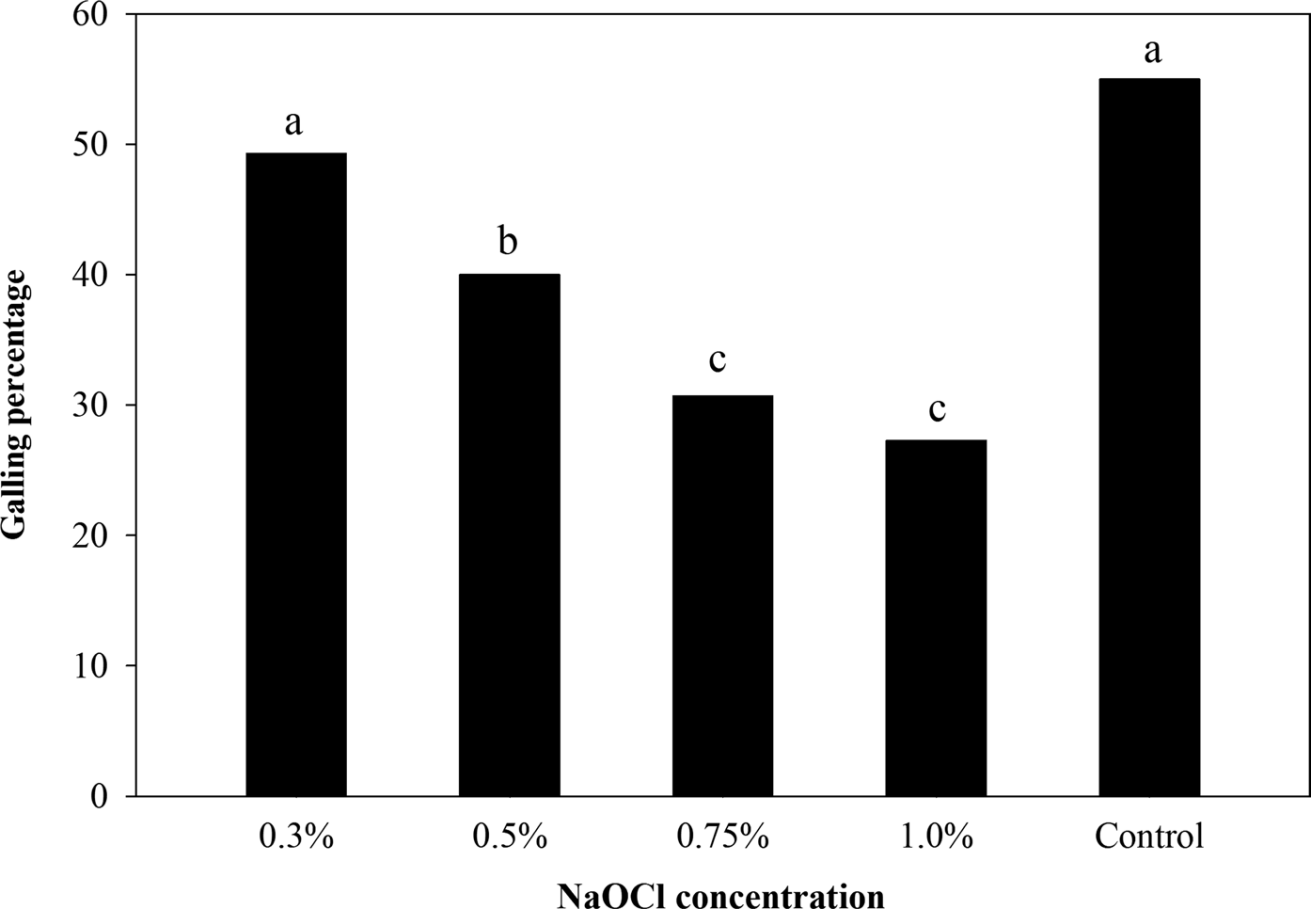
Effect of NaOCl concentrations on *M. enterolobii* infectivity on cucumber roots. Inoculum densities of 6,000 ± 60 eggs and 1,500 ± 15 J2 for control treatment. Data are means of 32 replications (16 replications/trial). Bars with the same letter are not significantly different (*p >* 0.05) by Fisher’s least significant difference (LSD) test.

At 5 WAI, despite significant differences in egg counts, galling levels were statistically inappropriate for analysis (data not shown). At 17 WAI, only the concentration of NaOCl was a significant factor in galling. Here, the treatment with 0.3% NaOCl resulted in higher (*p ≤* 0.05) percentages of galling when compared with the rest of NaOCl treatments (Fig. 5). The 0.75% and 1.0% NaOCl treatments showed the lower galling levels (*p ≤* 0.05). Control treatment of 1,500 ± 15 J2 resulted in a similar galling level when compared to 6,000 ± 60 eggs from the 0.3% NaOCl treatment at 17 WAI (*p >* 0.05) (Fig. 5).

## Discussion

To extract eggs of *Meloidogyne* spp., the galled roots can be directly treated with sodium hypochlorite via shaking, grinding or stirring to release the eggs from the gelatinous matrix attached to the root surface (Stetina et al., 1997). However, NaOCl potentially damages the egg structure upon contact. Consequently, NaOCl is also potentially detrimental to the larvae. Reduced viability of eggs extracted with NaOCl has been previously reported in comparison with J2 or egg masses excised without NaOCl treatment (Bird, 1968; Di Vito et al., 1986; McClure et al., 1973; Vrain, 1977). In our experiments, different NaOCl concentrations and stirring periods were used to extract *M. enterolobii* eggs. Here, the estimation of the egg population in the cucumber roots was achieved by several treatments. However, it was demonstrated that the extraction level of *M. enterolobii* eggs was reduced when combining low concentrations of NaOCl with brief stirring periods. Also, eggs were disintegrated when combining high concentrations of NaOCl with long stirring periods.

During the embryonic development of *Meloidogyne* spp., eggshell affords protection against toxic compounds through the inner lipid membrane, which is degraded via enzyme activity immediately prior to the hatch of the J2 (Curtis et al., 2009). A hatching delay or reduction of infective second-stage juveniles has been previously described with NaOCl concentrations from 0.5 to 1.0% (Di Vito et al., 1985, 1986; Vrain, 1977). In our results, it was proved that instead a hatching delay a higher hatching was induced by effect of the increment in the NaOCl concentration or in the stirring period. Unfortunately, the higher induced hatching resulted in most of larvae incapable to survive and infect the cucumber roots, this was partially demonstrated by the negative correlation between hatching and reproduction data.

While all the 0.1 and 0.75% NaOCl treatments observed higher levels of hatching, only the 0.7% NaOCl with 8 min stirring also showed a higher number of produced eggs compared with the 0.3% NaOCl treatments (within each stirring period). The above means that despite the negative relation between hatching and egg production, a moderate induction of hatching resulted in a faster infection. In the same way, it is known that there is a relation between the number of RKN infecting roots and the size of galls (Vovlas et al., 2005b). In our experiments at 17 WAI, the higher galling level corresponded to the treatment with the lower NaOCl concentration. This indicated that the 0.3% NaOCl treatment resulted in a higher number of nematodes infecting the roots. Therefore, a higher number of larvae survived with the 0.3% NaOCl treatments when compared with the 0.5, 0.75, and 1.0% NaOCl treatments. As mentioned above, both hatching and reproduction levels (at 5 WAI) from treatment of 0.3% NaOCl with 8-min stirring were lower when compared with those from 0.75% NaOCl with 8-min stirring treatment. Here, this evidences that the hatching process was unaffected by the 0.3% NaOCl concentration. So, the lower egg production (observed at 5 WAI) despite the higher number of nematodes infecting the roots (demonstrated at 17 WAI) was caused by the natural asynchrony in biological development of larvae inside the eggs. In turn, hatching and invasion processes (from eggs extracted with 0.3% NaOCl with 8-min stirring) occurred in a broader period of time, compared with the rest of treatments.

We conclude that the effect of the NaOCl concentration on the viability of *M. enterolobii* eggs is potentially detrimental. However, it is achievable to obtain both a reliable estimation of egg population in roots and a viable inoculum. The combinations of 0.5% NaOCl with stirring for 8, 12, and 16 min, and 0.75% NaOCl for 8 min are preferable to extract the eggs of *M. enterolobii.* The NaOCl concentrations from 0.5 to 0.75% (with 8-min stirring) are more appropriate to induce a faster hatching of viable larvae. Finally, we conclude that the 0.3% NaOCl concentration is preferable to extract *M. enterolobii* eggs with more viability.
